# Cleft Lip and Palate

**Published:** 2013-01-30

**Authors:** John Pang, Justin Broyles, Richard Redett

**Affiliations:** The Department of Plastic and Reconstructive Surgery, The Johns Hopkins Hospital, Baltimore, Md

**Figure:**
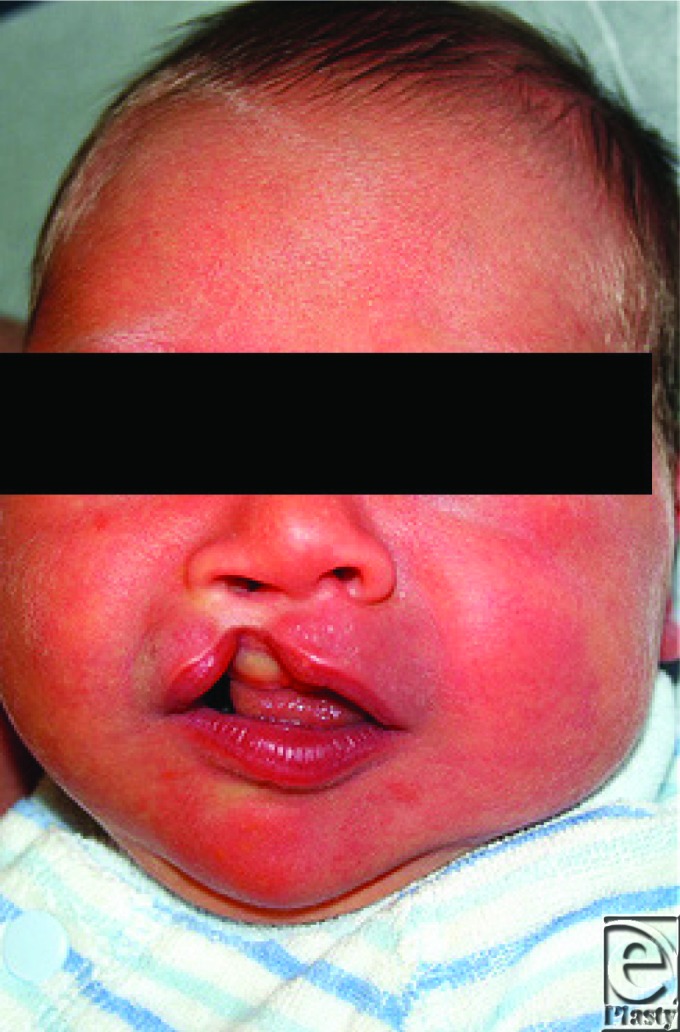


## DESCRIPTION

A 2-week-old infant with unilateral cleft lip and palate (CLP) is brought to clinic by his parents. He was born full-term and the only associated abnormality is a small atrial septal defect. There is no family history of clefting.

## QUESTIONS

1. **Discuss the embryologic basis of clefting, as well as the epidemiology of cleft lip alone and cleft palate with or without cleft lip. What environmental factors cause or reduce clefting?**
2. **What is the relationship between cleft anomalies and otologic problems?**
3. **Discuss the optimal time to repair cleft lip.**
4. **Discuss several approaches to repairing cleft palate and their advantages/disadvantages.**
5. **What clinical variables increase the risk of postoperative fistulas? How soon after the initial surgery should the fistula be repaired?**

## DISCUSSION

With an estimated incidence of approximately 1 per 700 live births, CLP is quite common. Cleft lip and palate is the most common diagnosis (46%), followed by cleft palate alone (33%) and then cleft lip alone (21%).^1^ Approximately 30% of clefts occur as part of a genetic syndrome, and these cases tend to be either cleft lip alone or cleft palate alone. Less than 15% of CLP cases are associated with malformation syndromes.^2^

Embryologic development of the lip and palate is a complex process that begins at approximately 4 weeks' gestation. The medial nasal processes contact the maxillary processes to form the primary palate. Secondary palatal shelves develop as bilateral outgrowths from the maxillary processes and eventually fuse together with the primary palate anteriorly, dividing the oronasal space into oral and nasal cavities. Unilateral and bilateral CLP is nearly always due to failure of the medial nasal processes to contact the maxillary process. Occasionally, a fibrous band (known as Simonart's band) is observed across or attached the cleft, suggesting that occlusive epithelial adhesions can result in CLP.^2^

More rarely, medial CLP can occur if the 2 medial nasal processes fail to fuse in the midline. Oblique facial clefts result from failure of the lateral olfactory placode to fuse with the medial maxillary process. Lateral facial clefts result from failure of the maxillary and mandibular processes to fuse. Median mandibular clefts result when the medial ends of the first pharyngeal arch fail to fuse, possibly because of abnormal growth centers at the arch's medial tips.^3^

Maternal smoking and alcohol consumption have been associated with a higher incidence of CLP. Folate deficiency is also associated with CLP, and supplemental doses (6 mg/d) have been shown to decrease the risk in some studies. The folic acid antagonist and antiepileptic phenytoin increase the incidence 10-fold. Maternal corticosteroid use causes a 3-fold increase in CLP.^2^^,^^3^

Up to 70% of young children with cleft palate suffer from otitis media with effusion and a hearing loss greater than 20 dB.^4^ This may be secondary to Eustachian tube dysfunction leading to dysregulation of middle ear pressure. While children without cleft palate tend to overcome otologic difficulties after the age of 6 years, those with cleft palate tend to be symptomatic up to 12 years of age. Approximately one-third of patients require tympanostomy tubes and 8% require tympanoplasty and/or mastoidectomy.^5^

Prophylactic tympanostomy tube insertion at the time of CP repair has not been shown to produce long-term benefits in speech or language development. Moreover, studies on patients with CP have shown that their otologic problems dramatically decrease after the age of 10 years, so their long-term otologic outcome is quite good.^5^

Historically, the optimal time for cleft lip repair is accepted to be 10 weeks of age due to the increased risk of postoperative complications during the neonatal period. However, there have been major advances in neonatal care since this view was first established in the mid-1960s, including pediatric airway management, anesthesiology, and postoperative care. Recently, several studies have demonstrated that neonatal repair of full-term neonates is not unsafe. In a retrospective study of 99 CLP patients repaired within 28 days of birth, there were 5 cases of postoperative complications, all for airway-related issues that were easily managed by a pediatric anesthetist and PICU.^6^ Possible benefits of early-repair include improved feeding and subsequent benefit for the parent-child relationship, as well as improved cosmesis. However, a comparison of 29 early repair patients and 34 late repair patients found no significant difference in cosmesis.^7^

For patients with clefting of the hard palate, the optimal time to repair the palate is recommended by most sources to be before 18 months. Later repair of the hard palate results in improved anterioposterior development of the maxillary dentoalveolus, and hard palate repair after pubertal peak velocity was shown to result in less forward displacement of the maxilla.^8^ However, delaying repair beyond 3 years of age severely restricts language development and produces stereotyped cleft speech characteristics.

There are several approaches to repairing the cleft palate. Three commonly used approaches are the von Langenbeck procedure, the Bardach 2-flap palatoplasty, and the Furlow double-opposing Z-plasty.

The von Langenbeck procedure is a simple palatal closure. Incisions are made along the cleft edges and the posterior margin of the alveolar ridge. Bipedicled mucoperiosteal flaps are then raised and joined in the midline medially. The main advantages of the von Langenbeck procedure are its simplicity and the relatively little amount of dissection required to perform it. Disadvantages include a greater risk of anterior fistulas as well as inferior speech due to the insufficiently long soft palate.

The Bardach 2-flap palatoplasty is a modified von Langenbeck procedure. Modifications include dissecting the levator veli palatini muscles off the hard palate to improve the medial rotation of the mucoperiosteal flaps, reducing tension. This reduces the incidence of fistula formation to as low as 3.4%. In addition, a muscle sling is created within the soft palate to improve velopharyngeal function and speech quality.

The Furlow double-opposing Z-plasty is growing in popularity. The Z-plasty on the patient's left creates a flap of oral mucosa containing the underlying muscle, whereas the Z-plasty on the patient's right creates a flap of oral mucosa only (the muscle is left attached to the nasal mucosa). The 2 muscle-bearing flaps are transposed posteriorly, while the non–muscle-bearing flaps are transposed anteriorly, creating a lengthened velum and a functioning levator muscle sling. The Z-plasty produces excellent outcomes with regard to speech development, maxillary development, and speech development. However, it is difficult to perform in wide clefts, so some authors have recommended its use for clefts smaller than 8-mm wide and the Bardach repair for wider clefts.

Postoperative palatal fistulas are not uncommon, with an incidence reported to be from 3% to 30%. Most commonly located at the junction of the hard and soft palate, fistulas may cause the patient to develop articulation difficulties and food regurgitation into the nose. Cleft size correlates with the risk of fistula development. In general, palatal fistulas should not be closed within 6 months of the initial surgery. Within this time period, the blood supply to the local tissue can be tenuous. Therefore, it is preferable to wait until 6 to 12 months after the initial surgery to repair the fistula. Buccal and tongue flaps may be used to close the fistula if there is inadequate local tissue adjacent to the fistula for simple closure.
